# Purinergic signaling in inflammatory renal disease

**DOI:** 10.3389/fphys.2013.00194

**Published:** 2013-07-29

**Authors:** Nishkantha Arulkumaran, Clare M. Turner, Marije L. Sixma, Mervyn Singer, Robert Unwin, Frederick W. K. Tam

**Affiliations:** ^1^Imperial College Kidney and Transplant Institute, Imperial College London, Hammersmith HospitalLondon, UK; ^2^Division of Medicine, Bloomsbury Institute of Intensive Care Medicine, University College LondonLondon, UK; ^3^Division of Medicine, UCL Centre for Nephrology, Royal Free Campus and Hospital, University College LondonLondon, UK

**Keywords:** purines, inflammation, renal circulation, ischemia, renal disease

## Abstract

Extracellular purines have a role in renal physiology and adaption to inflammation. However, inflammatory renal disease may be mediated by extracellular purines, resulting in renal injury. The role of purinergic signaling is dependent on the concentrations of extracellular purines. Low basal levels of purines are important in normal homeostasis and growth. Concentrations of extracellular purines are significantly elevated during inflammation and mediate either an adaptive role or propagate local inflammation. Adenosine signaling mediates alterations in regional renal blood flow by regulation of the renal microcirculation, tubulo-glomerular feedback, and tubular transport of sodium and water. Increased extracellular ATP and renal P2 receptor-mediated inflammation are associated with various renal diseases, including hypertension, diabetic nephropathy, and glomerulonephritis. Experimental data suggests P2 receptor deficiency or receptor antagonism is associated with amelioration of antibody-mediated nephritis, suggesting a pathogenic (rather than adaptive) role of purinergic signaling. We discuss the role of extracellular nucleotides in adaptation to ischemic renal injury and in the pathogenesis of inflammatory renal disease.

## Introduction

Purines are ubiquitous molecules that are synthesized as nucleosides. There are several purines with wide-ranging functions. Adenine and guanine are purines that are essential for the synthesis of DNA and RNA. Adenosine is an endogenous purine nucleoside that comprises a molecule of adenine linked to a ribose sugar molecule. As a component in cyclic adenosine monophosphate (cAMP), adenosine plays a major role in signal transduction. Adenosine also provides energy for several intracellular biologic reactions, with adenosine 5′-triphosphate (ATP) and ADP as the principal energy sources. Other purines include xanthine, hypoxanthine, and uric acid.

Among the endogenous purinergic compounds, adenosine and ATP play a key role in the host response to inflammation via extracellular signaling pathways. Extracellular levels of adenosine and ATP are increased several-fold during inflammation, and act on specific purinergic receptors. Activation of adenosine receptors tends to mediate cytoprotection in response to inflammation, whereas activation of the ATP-sensitive P2X_7_ receptor triggers pro-inflammatory signaling. In this review, we discuss the physiological and pathological roles of purinergic signaling in inflammatory renal disease, with particular focus on adenosine receptors and ATP-sensitive P2 receptors.

## Extracellular purines in the kidney

Within the cytosol, ATP is present in millimolar concentrations (~3–5 mM in most cells). Much lower concentrations of ATP are found in the extracellular environment under basal conditions. Various physiological and pathological conditions are associated with elevated levels of extracellular nucleotides.

The relative contribution of different cell types to the basal levels of extracellular nucleotides and the mechanism of purine release is an area of active research. The most widely accepted mechanism of release is via cell lysis- either non-specific cytolysis of healthy cells by mechanical trauma or as a result of necrosis or apoptosis. An alternative mechanism involves exocytotic release of ATP and ADP via granules or vesicles that contain compartmentalized nucleotides. Exocytotic release of ATP via granules is well-described in neurons and neuro- endocrine cells (von Kugelgen et al., 1998; Bodin and Burnstock, 2001a; Fabbro et al., 2004). Non-neuronal cells of exocrine and endocrine origin store large amounts of ATP within cytosolic granules which are released into the extracellular environment (>100 mM) (Coco et al., 2003; Obermuller et al., 2005). Extracellular release of ADP via granules has also been described in activated platelets and immune cells (Eltzschig et al., 2006, 2008; Weissmuller et al., 2008).

The conductive release of ATP via intrinsic plasma membrane channels or pores in the absence of irreversible cytolysis is another proposed mechanism of extracellular ATP release. There are various classes of cell membrane channels associated with conductive release of ATP. Examples include pore forming P2X_7_ receptors (Ferrari et al., 1996), connexins (Kang et al., 2008) and pannexins (Huang et al., 2007), maxi-anion channels and volume-regulated ion channels (Hisadome et al., 2002; Bell et al., 2003, 2009).

The multiple sources of extracellular ATP have also been described for the kidney. ATP is co-released with acetylcholine or noradrenaline from nerve termini via synaptic vesicles within the kidney (Dowdall et al., 1974; Burnstock, 1995). Electrical field stimulation of the sympathetic nerves in the renal cortex induces release of ATP. However, this accounts for only 25% of the total cortical ATP content, with the remainder coming from non-neuronal sources (Vonend et al., 2002). Under basal conditions, nanomolar concentrations of ATP are released from proximal tubule cell primary cultures, cell lines, and *in vivo* (Schwiebert, 2001; Praetorius and Leipziger, 2010). Glomeruli have been shown to constitutively release ATP, and the macula densa releases ATP via maxi anion channels (Bell et al., 2003, 2009; Karczewska et al., 2007). Thick ascending limb and collecting duct cells have also been shown to release nanomolar concentrations of ATP (Schwiebert, 2001). Apart from epithelial cells, other potential sources of ATP in the kidney include cells of the vascular network such as endothelial and smooth muscle cells (Pearson and Gordon, 1979; Bodin and Burnstock, 1996, 2001b; Yamamoto et al., 2003), platelets (Detwiler and Feinman, 1973; Hechler and Gachet, 2011), mononuclear cells (Maugeri et al., 1990), and erythrocytes (Bergfeld and Forrester, 1992; Wan et al., 2011). The relative amounts of ATP vary within the kidney, with ATP concentration in the proximal tubules being 4-fold greater than in Bowman's space (Vekaria et al., 2006). Cellular release of ATP can be induced by a range of stimuli, including hypoxia (Bergfeld and Forrester, 1992), acute inflammation (Bodin and Burnstock, 1998), fluid shear stress (Bodin and Burnstock, 2001b), osmotic shock (Jans et al., 2002), and mechanical deformation such as distension of the ureter (Knight et al., 2002). Once released from cells, ATP may influence cells in an autocrine or paracrine fashion. Extracellular ATP and other nucleotides are rapidly hydrolyzed by membrane-bound ecto-enzymes such as ecto-5′-nucleotidase (Dawson et al., 1989). Therefore, P2 receptor signaling in the kidney involves a balance between ATP release and ATP breakdown.

Basal levels of adenosine are in the range of 300 nM, but may increase up to 4-fold during acute inflammation. Extracellular adenosine is generated from the phosphohydrolysis of extracellular ATP and ADP by constitutive ectonucleoside-triphosphate-diphosphohydrolase-1 (CD39) and ecto-5′-nucleotidase (CD73). CD39 catalyzes the breakdown of extracellular ATP and ADP by rapid conversion to AMP, whereas CD73 is a membrane-bound protein responsible for the final step in generating extracellular adenosine by converting AMP to adenosine. Alternatively, extracellular adenosine may be released by circulating cells such as activated immune cells and platelets (Bauerle et al., 2011).

## Purinoceptors in the kidney

**Figure 1:**
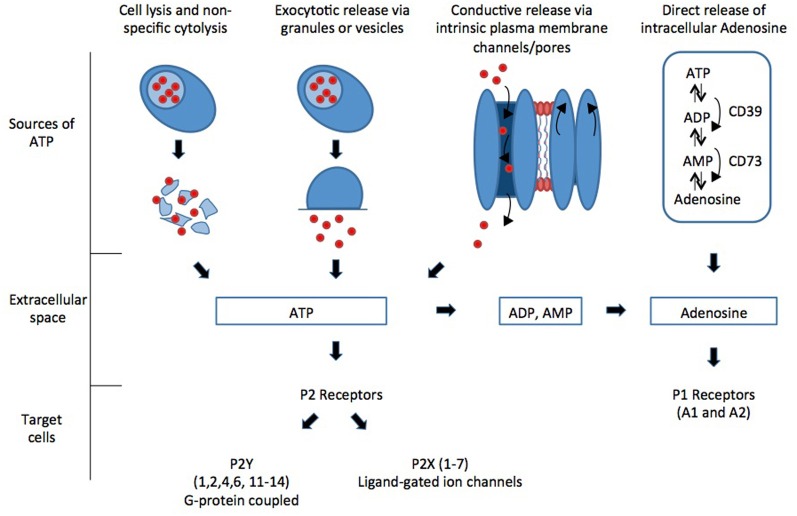
**Intracellular purines are released into the extracellular space by various mechanisms and act upon extracellular purinergic receptors**.

Purinergic signaling occurs via activation of specific receptors called purinergic receptors. Purinergic receptors can be divided into two main families, the P1 receptors that are selective for adenosine and the P2 receptors that are selective for ATP and ADP. Intracellular purines are released into the extracellular space by various mechanisms and act upon extracellular purinergic receptors (Figure 1).

### P1 receptors

The effects of extracellular adenosine are mediated via G-protein coupled purinergic receptors. These include A1, A2A, A2B, and A3 receptors. Adenosine receptors can be subdivided into those that are activated by physiological levels only of adenosine (10–100 nM) and those that require much higher levels of adenosine (>1 μM), as seen in pathological conditions (Fredholm, 2007). A1, A2A, and A3 receptors require physiological levels of adenosine, whereas A3 receptors require much higher concentrations of adenosine for activation. The A1 (high affinity) and A3 (low affinity) receptors mediate inhibition of the enzyme adenylyl cyclase (AC) and inhibit formation of 3′, 5′-cyclic adenosine monophosphate (cAMP), whereas the A2A (high affinity) and A2B (low affinity) receptors stimulate AC.

The A1AR is widely expressed in the kidney, especially in the afferent arterioles, mesangial cells, proximal convoluted tubules, medullary collecting ducts, and papillary epithelia. The A2AAR receptor is located predominantly in the glomerular epithelium and adjacent vasculature (Bauerle et al., 2011). The A2BAR receptors are found predominantly in the renal vasculature with minimal expression in renal epithelia. The precise location and role of the A3 receptor within the kidney remains unclear. The role of renal adenosine receptors centers primarily on regulation of renal vascular tone and glomerular filtration rate (GFR).

### P2 receptors

The P2 purinergic receptors can in turn be divided into two subcategories: the inotropic P2X (ligand-gated ion channels) and metabotropic P2Y receptors (G-protein-coupled receptors). To date, seven P2X subunits (P2X1-7) and eight P2Y subunits (P2Y1,2,4,6,11–14) have been identified. The effect of extracellular ATP on renal epithelial cells was first reported in 1972, when ATP was shown to increase calcium release from a suspension of cortical tubules (Rorive and Kleinzeller, 1972). ATP is now known to exert its effects via distinct P2 receptors. P2 receptor mRNA and protein are expressed throughout the nephron, vasculature, and interstitial cells (Bailey et al., 2000, 2001; Turner et al., 2003). Differential basolateral and apical cell membrane expression of P2 receptors has been reported (Bailey et al., 2000, 2001; Deetjen et al., 2000; Kishore et al., 2000; Turner et al., 2003). The roles postulated for P2 receptors in the kidney are multiple and diverse, including cell cycle regulation, mediators of hormonal control in the proximal tubule, mediators or moderators of TGF, and regulators of ion transport processes along the nephron. Numerous studies have shown that ATP contributes to many pathophysiological processes, including cell proliferation, apoptosis, vascular remodeling, inflammation and necrosis.

One of the key purinoceptors in promoting inflammation is the P2X_7_ receptor (P2X_7_R). Expression of this receptor was originally defined in macrophages and monocytes (Di Virgilio et al., 2001), and has since been characterized in several different cell types, including intrinsic renal cells (Arulkumaran et al., 2011). Unlike other P2X receptors the P2X_7_ receptor is distinct, because its downstream signaling is coupled to pro- inflammatory cascades (North, 2002). Cell damage and death during active inflammation results in an increase in local concentrations of extracellular ATP. Activation of the P2X_7_R *in vitro* requires extracellular concentrations of ATP in the range of 1 mM, in contrast to concentrations <100 μM needed to activate other P2 receptors. Therefore, the P2X_7_ receptor is thought to act as a “danger signal” in response to damaged or lysed cells. Transient activation of the P2X_7_R leads to release of the pro-inflammatory cytokines IL-1β and IL-18. Prolonged activation of P2X_7_R results in irreversible pore formation and allows the non-selective passage of ions and hydrophilic solutes of up to 900 Da, which can result in colloido-osmotic lysis and cell death by apoptosis or necrosis (Ferrari et al., 2006).

## Purines and cell survival

P2 purinoceptos have been shown to regulate cell survival and death. The precise functional effect of purinoceptors on cell survival is very much dependent on the local environment. Studies measuring the incorporation of ^3^H-thymidine into the cellular DNA of mesangial cells after agonist stimulation of P2 receptors have demonstrated that extracellular nucleotides can regulate mesangial cell proliferation *in vitro* via P2Y receptor activation (Schulze-Lohoff et al., 1992; Huwiler and Pfeilschifter, 1994; Ishikawa et al., 1994; Harada et al., 2000). More recently, ATP-induced proliferation of mesangial cells was shown to be through P2Y receptor activation of the MAPK^42/44^ signal transduction pathway (Vonend et al., 2003). Extracellular nucleotides may also activate the stress-activated protein kinase (SAPK) and the p38-stress-activated protein kinase (MAPK^38^) cascades in mesangial cells via P2Y receptors (Huwiler et al., 1997, 2000). These cascades are most often activated by cellular stresses such as chemicals, heat, osmotic shock, and UV irradiation (Paul et al., 1997). The balance between nucleotide-stimulation of the MAPK^42/44^ pathway (Vonend et al., 2003), the SAPK and MAPK^38^ pathways, determines whether cells survive or undergo apoptosis (Huwiler et al., 1997, 2000). Cultured mesangial cells have been shown to undergo apoptosis via stimulation of the P2X_7_R by ATP and inhibited by P2X_7_ receptor antagonists (Schulze-Lohoff et al., 1998). The trigger for apoptosis has most often been associated with P2X_7_R activity (Schulze-Lohoff et al., 1998; Bulanova et al., 2005; Turner et al., 2007; Taylor et al., 2009; Kawano et al., 2012) although P2X_4_R-mediated apoptosis has been reported in cultured human mesangial cells and in a mouse macrophage cell line (Kawano et al., 2012; Solini et al., 2007).

Interestingly a proliferative role has also been suggested for the P2X_7_ receptor in non-renal cells. P2X_7_ transfected HEK293 cells exhibited enhanced tumorigenesis when inoculated into immunodeficient BALB/c mice (Adinolfi et al., 2012); moreover, P2X_7_R expression has been reported to be upregulated in malignant tumor cells (Di Virgilio et al., 2009).

## Inflammatory renal diseases

### Renal ischemia

During global hypoxia or impaired renal perfusion, ATP consumption exceeds ATP production. This results in elevated adenosine levels (Miller et al., 1978; Beach et al., 1991; Nishiyama et al., 1999). This may be seen in a number of acute clinical scenarios, including hemorrhage and endotoxaemia. Adenosine signaling mediates alterations in regional renal blood flow, preserving bioenergetics and cellular structure (Weinberg and Venkatachalam, 2012).

Tubulo-glomerular feedback (TGF) is a negative feedback mechanism whereby changes in luminal NaCl delivery and reabsorption at the macula densa can adjust the vascular tone of the afferent arteriole to alter and match glomerular filtration (Schnermann et al., 1973). TGF allows each nephron to regulate its GFR in accordance with the capacity of its proximal tubule to reabsorb NaCl. Extracellular adenosine contributes to the regulation of GFR. Renal interstitial adenosine is mainly derived from dephosphorylation of released ATP, AMP, or cAMP by the enzyme ecto-5′-nucleotidase (CD73) (Le Hir and Kaissling, 1993). This enzyme catalyzes the dephosphorylation of 5′-AMP or 5′-IMP to adenosine or inosine, respectively, and is located primarily on the external membranes and mitochondria of proximal tubule cells, but not in distal tubule or collecting duct cells (Miller et al., 1978). ATP consumed in active transport by the macula densa also contributes to the formation of adenosine by 5- nucleotidase (Thomson et al., 2000). Extracellular adenosine activates A1 receptors on vascular afferent arteriolar smooth muscle cells, resulting in vasoconstriction and a reduction in GFR (Schnermann et al., 1990). Renal clearance and micropuncture experiments in CD73 null mice demonstrate a diminished TGF response to increasing proximal tubule perfusion (Huang et al., 2006). These effects of extracellular adenosine are mediated via A1 receptors, since blockade of these receptors completely abrogates TGF, and mice deficient in adenosine A1 receptors also lack a normal TGF response (Vallon et al., 2004). P2X1 receptors autoregulate renal blood flow when juxtamedullary afferent arteriolar pressure is elevated. Activation of P2X_1_ receptors on preglomerular arteriolar smooth muscle cells results in an increase in intracellular Ca^2+^ concentration, leading to vasoconstriction. This response is blunted in hypertensive (Angiotensin II treated, high- salt diet) rats, suggesting that P2X_1_ receptors are important for pressure-mediated autoregulatory responses (Inscho et al., 2011).

Elevated extracellular concentrations of adenosine within the kidney are seen in local ischemia (Miller et al., 1978; Beach et al., 1991; Nishiyama et al., 1999). During ischemia, aerobic metabolism and ATP production are impaired. This limits the ability of the proximal tubule to reabsorb filtered electrolytes and water, a highly energy-dependent process. Elevated adenosine levels may limit GFR via TGF, reducing the need for tubular reabsorption and preserving renal bioenergetics. Experimental work demonstrates that generation of adenosine by renal CD73 during renal ischemia, and selective renal A1 receptor agonists protect against renal ischemia and reperfusion injury (Park et al., 2012; Kim et al., 2013).

Renal concentrations of adenosine are elevated in response to endotoxic shock (Miller et al., 1978; Beach et al., 1991; Nishiyama et al., 1999). In an experimental model of endotoxaemia and shock, reduced mean arterial pressure and renal blood flow were associated with an elevated renal adenosine concentration. Antagonism of the A1 receptor attenuated the reduction in mean arterial pressure and renal blood flow, suggesting that adenosine may preserve renal blood flow during endotoxic shock (Nishiyama et al., 1999).

Selective augmentation of adenosine around afferent arterioles causes persistent vasoconstriction, indicating A1AR dominance. In contrast to the A1AR-receptor, the A2AAR-receptor mediates vasodilatation in deep cortical glomerular vessels, and increases medullary blood flow and oxygenation (Vallon and Osswald, 2009). Global elevation of renal adenosine causes steady-state vasodilatation from A2AR-mediated generation of nitric oxide (Hansen et al., 2005). Thus, global increments in adenosine production and release may increase total blood flow through A2AR generation of NO, while allowing a compensatory reduction in tubular workload by a reduction in A1AR activation. This can maintain renal oxygenation, while at the same time limiting renal workload. Adenosine protects from ischemic acute kidney injury (AKI) in a mouse model of ischemia -reperfusion by preserving peritubular capillary blood flow during reperfusion via activation of A2AR on endothelial cells (Grenz et al., 2012).

### Vascular remodeling and hypertension

Immunohistochemistry has shown that P2X_1_, P2X_2_, P2X_7_ and P2Y_1_ receptors are expressed on the renal vasculature (Lewis and Evans, 2001; Turner et al., 2003) and are responsible for the regulation of renal hemodynamics (reviewed in Guan et al., 2007). Stimulated endothelial cells release endogenous nucleotides and changes in renal arterial pressure result in increased interstitial ATP concentration (Yegutkin et al., 2000; Nishiyama et al., 2001). Studies indicate that alteration in ATP/P2 receptor signaling may result in renal vascular dysfunction and may lead to hypertension. Angiotensin II mediates hypertension by promoting mesangial cell transformation, renal inflammation, and vascular hypertrophy. Mice deficient in P2X_1_ receptors or treated with the non-specific P2 receptor antagonist PPADS, or a specific P2Y_12_ receptor antagonist, demonstrate an attenuation of angiotensin II-mediated hypertension (Graciano et al., 2008; Franco et al., 2011). This suggests that angiotensin II mediates hypertension via its action on vascular and glomerular P2 receptors. P2Y_2_ and P2Y_4_ receptors regulate ENaC activity in the collecting duct, thereby regulating sodium balance and systemic hypertension (Wildman et al., 2008). Glomerular hypertension results in glomerular capillary wall stretch, endothelial damage, and a rise in protein glomerular filtration, leading to further ATP release (Bodin and Burnstock, 1998; Yegutkin et al., 2000; Nishiyama et al., 2001; Schwiebert, 2001). This autocrine loop of released ATP and cell stimulation exacerbates the inflammatory response by further release of inflammatory cytokines, as supported by a rat model of renin-dependent hypertension in which glomerular P2X_7_R was up-regulated at 12 weeks (Vonend et al., 2004). The P2X_4_ receptor has also been shown to regulate vascular remodeling. Local ATP released during increased renal blood flow acts upon endothelial P2X_4_ receptors, resulting in calcium influx and the production of nitric oxide. This effect is attenuated in P2X_4_-deficient mice, and, P2X_4_-deficient mice fail to exhibit adaptive vascular remodeling in response to a chronic decrease in renal blood flow (Yamamoto et al., 2006).

### Antibody mediated renal disease

Increased glomerular P2X_7_R expression has been reported in a mouse model of accelerated nephrotoxic nephritis, with P2X_7_R expression coinciding with an increase in apoptotic cells (Turner et al., 2007). Similarly, in a rat model of antibody-mediated glomerulonephritis increased P2X_7_R mRNA expression coincided with the onset of proteinuria and an increase in IL-1β and pro-apoptotic genes (Turner et al., 2007). P2X_7_R is also upregulated in patients with lupus nephritis. Genetic deletion of P2X_7_R is associated with amelioration of renal injury in a murine model of antibody-mediated glomerulonephritis, with reduced macrophage infiltration, less fibrin deposition, less glomerular thrombosis, reduced thrombosis and overall protection of renal function. This coincides with a reduction in the chemokine CCL2 in P2X_7_^−/−^ mice compared with wildtype controls (Taylor et al., 2009). Similarly the P2X_7_R antagonist A438079 reduces the severity of antibody-mediated glomerulonephritis in rats with reduced proteinuria and renal macrophage infiltration (Taylor et al., 2009). In a model of passive crescentic glomerulonephritis, P2Y_1_ gene deficiency was also renoprotective, reducing capillary loss, fibrosis, and death by renal failure (Hohenstein et al., 2007). The current evidence are based on models induced by passive injection of glomerular binding antibodies. The roles of purinergic receptors in antigen presentation and development of autoimmune response need to be investigated.

### Diabetic kidney disease

Significant glomerular expression of P2X_7_R is reported in rats with steptozotocin-induced diabetes with a corresponding 10-fold increase in P2X_7_R mRNA expression (Vonend et al., 2004). Electron microscopy demonstrated that P2X_7_R was located primarily on podocytes and endothelial cells. Both oxygen and glucose deprivation have been shown to increase integration of P2X_7_R into the cell membrane of HEK 293 cells (Milius et al., 2007). Culturing cells in high glucose-containing media stimulated ATP secretion in rat mesangial cells, but with no change in P2 receptor expression (Solini et al., 2005). High glucose has also been shown to initiate inflammasome activation through P2X_4_R regulation in HK-2 cells (Chen et al., 2013). In type 2 diabetic patients with nephropathy, tubular P2X_4_R expression was upregulated and closely related to NLRP3 inflammasome activation and renal interstitial inflammation (Chen et al., 2013). The functional importance of purinergic receptors in diabetic nephropathy still require further investigation *in vivo*, and in clinical studies.

### Ureteric obstruction

The role of P2X_7_R and TGF-β in extracellular matrix deposition was demonstrated in a mouse model of unilateral ureteral obstruction (UUO) (Goncalves et al., 2006). Myofibroblast number and collagen deposition were significantly reduced in P2X_7_R KO mouse compared with wildtype mice in a UUO model. Immunohistochemistry demonstrated significantly less Myofibroblasts and Sirius-red staining in KO mice on day 7 with a reduction in TGF-β. Furthermore, at day 14, there were reduced numbers of infiltrating macrophages associated with less tubular cell apoptosis in kidneys from the P2X_7_R KO mice. The tubulo-interstitial damage and subsequent fibrosis at this time point were attenuated in P2X_7_R KO mice, again implicating tubular P2X_7_R in renal inflammation and fibrosis.

## Conclusion

Purines play a key role in maintaining renal physiology during health and mediate adaptation to pathologic conditions, including ischemia and endotoxaemia. P2 purinoreceptors are “danger sensors” and are key players in inflammation. However, in certain circumstances, inflammation mediated via P2 receptors may become pathological, leading to tissue damage and renal injury. The potential role of selective antagonists against purinoreceptors as a therapeutic target in pro-inflammatory renal diseases, including glomerulonephritis and diabetes requires further evaluation.

### Conflict of interest statement

Nishkantha Arulkumaran, Wellcome Trust pre-doctoral training fellowship. Marije Sixma, Dutch kidney foundation, Kolff student grant. Robert J. Unwin, Consultancy with AstraZeneca. Frederick Tam, Research project grants from Roche Palo Alto, AstraZeneca Limited, Cyclacel Limited and Baxter Biosciences, Consultancy for Roche Palo Alto and Baxter Biosciences. Clare Turner and Mervyn Singer declare that the research was conducted in the absence of any commercial or financial relationships that could be construed as a potential conflict of interest.
